# Changes in Brain Size during the Menstrual Cycle

**DOI:** 10.1371/journal.pone.0014655

**Published:** 2011-02-04

**Authors:** Georg Hagemann, Tarik Ugur, Ekkehard Schleussner, Hans-Joachim Mentzel, Clemens Fitzek, Otto W. Witte, Christian Gaser

**Affiliations:** 1 Hans Berger Clinic for Neurology, University Hospital Jena, Jena, Germany; 2 Department of Neurology, HELIOS Klinik Berlin-Buch, Berlin, Germany; 3 Department of Psychiatry and Psychotherapy, Rheinische Kliniken Essen, University Duisburg-Essen, Essen, Germany; 4 Department of Obstetrics, Friedrich-Schiller-University, Jena, Germany; 5 Institute of Diagnostic and Interventional Radiology, Friedrich-Schiller-University, Jena, Germany; 6 Asklepios Fachklinikum Brandenburg, Institute of Radiology and Neuroradiology, Brandenburg, Germany; 7 Department of Psychiatry, Friedrich-Schiller-University, Jena, Germany; Baylor College of Medicine, United States of America

## Abstract

**Background:**

There is increasing evidence for hormone-dependent modification of function and behavior during the menstrual cycle, but little is known about associated short-term structural alterations of the brain. Preliminary studies suggest that a hormone-dependent decline in brain volume occurs in postmenopausal, or women receiving antiestrogens, long term. Advances in serial MR-volumetry have allowed for the accurate detection of small volume changes of the brain. Recently, activity-induced short-term structural plasticity of the brain was demonstrated, challenging the view that the brain is as rigid as formerly believed.

**Methodology/Principal Findings:**

We used MR-volumetry to investigate short-term brain volume changes across the menstrual cycle in women or a parallel 4 week period in men, respectively. We found a significant grey matter volume peak and CSF loss at the time of ovulation in females. This volume peak did not correlate with estradiol or progesterone hormone levels. Men did not show any significant brain volume alterations.

**Conclusions/Significance:**

These data give evidence of short-term hormone-dependent structural brain changes during the menstrual cycle, which need to be correlated with functional states and have to be considered in structure-associated functional brain research.

## Introduction

There is a vast amount of evidence for hormone-dependent modification of function and behavior during the menstrual cycle, but little is known about associated structural alterations of the brain [1], [2], [3], [4], [5]. Modern MR-techniques not only are capable to elucidate inter-individual brain atrophy during senescence or degenerative processes, but recently have also allowed for the discernment of short-term structural alterations induced by training [6]. This is evidence that the structure of the adult brain is not as rigid as formerly believed and can be visualized in humans. The expanding possibilities with respect to *in vivo* investigation into brain structure make it mandatory for us to increase our understanding of the physiological parameters affecting brain morphology. Both the function as well as the structure of the human brain are strongly dependent on sex and may be influenced by genetic as well as hormonal factors [2], [7], [8], [9], [10]. The hormonal influence on brain function and behavior is caused by developmental organizational effects and later on through modification and activation-related effects during maturation and adulthood [4], [7], [8], [11], [12], [13]. Furthermore, there are preliminary data demonstrating a long-term decline in brain volume in postmenopausal women or in women receiving antiestrogens, which can be prevented by hormone replacement therapy [14], [15], [16]. This finding suggests that estrogens may exert a measurable neuroprotective effect on the brain. Experimental work on rodents demonstrated that there are estrous dependent synaptic rearrangements in several brain regions which are functional significant and probably could be the underlying mechanism of gross morphological brain changes [5], [17].

Here we investigated, non-invasively, short-term brain volume changes during the menstrual cycle in women or a parallel 4 week period in men, respectively.

## Methods

### Subjects

Sixteen healthy volunteers (8 females and 8 eight males) were included in the study. The study protocol was approved by the local ethics committee (Ethik-Kommission der Friedrich-Schiller-Universität Jena), and all subjects gave written informed consent. Confounding comorbidity was excluded by an interview, with special emphasis on endocrine dysfunction and hypertension. No subjects were on medication, including hormonal contraceptives. All volunteers were asked to refrain from alcohol consumption the night before and coffee intake the morning before scanning. Each scanning session was scheduled early in the morning at the same time (7:30 am), with minimal food and fluid intake. Each male was paired with a female and scanned in one recording session at three or four time points during the respective menstrual cycle. Scanning took place during menses (t1), at time of ovulation (t2), and in the midluteal phase (t3). Half of the subjects were scanned again at the beginning of the next menses (t4). Only women known to have an ovulational cyclus were allowed for the scanning protocol. This was achieved by an intravaginal ultrasound performed by an experienced gynecologist. To ascertain an ovulational cyclus and the time point of ovulation (t2) during the month the actual MR-scanning took place, ultrasound was repeated and scanning at t2 took place the day after a follicle ready for ovulation was detected. Furthermore, in women, blood samples were taken at each scanning session and serum levels of estradiol and progesterone were evaluated, while excluding other hormonal alterations (thyroid function, testosterone, cortisol).

### MRI

Image data were acquired on a 1.5 T Siemens Vision using a standard birdcage head coil with a T1-weighted fl3d Gradient Echo Sequence (GRE, T_R_ = 15 ms, T_E_ = 5 ms, α = 30 °, 192 slices, sagittal orientation, voxel size 1×1×1 mm^3^). To increase the signal-to-noise ratio and to minimize the effects of different head positioning, subjects were scanned twice at each recording session with the subject exiting the scanner between scans. We used a longitudinal design with several repeated measures as this is much more powerful than a simple cross-sectional design with only one measure per subject [18], [19]. Data were visually checked for artifacts and any structural pathology.

### Data analysis

Data pre-processing and analysis was performed using SPM2 (Wellcome Department of Cognitive Neurology, London, UK). First, all scans of each subject were registered to the first baseline scan to correct the position of each scan without changing brain size. The first scan of each subject was then used to align this image to the default SPM2-template. The estimated alignment parameters of this process were finally applied to all other scans of each subject, to assure that the overall correction in brain size was the same for each subject, while the position was corrected individually. All images were partitioned into grey and white matter (GM/WM), cerebrospinal fluid (CSF) and background using a modified mixture model cluster analysis, after correcting for non-uniformities in image intensity [20]. In order to remove unconnected non-cerebral voxels, we applied a series of morphological erosions and dilations to the segmented images. To calculate tissue volume, we summed up the values for each compartment over the whole brain. Statistical analysis was performed by testing the interaction between sex and time for the time points t2-t4. We used an AnCova model with baseline values (t1) as nuisance parameter to control for the different brain size between females and males. The AnCova model was chosen because of the higher statistical power compared to the use of percentage change from baseline [21]. In females, we additionally tested the hypothesis that the relative volume changes were correlated with hormone levels. As the estradiol peak is a marker for the time of ovulation, correlations were calculated for volume-differences at time point t2 versus t1. Progesterone dominates the second half of the menstrual cycle, therefore, correlations were calculated for volume-differences at time point t3 versus t1.

## Results

**Figure 1:**
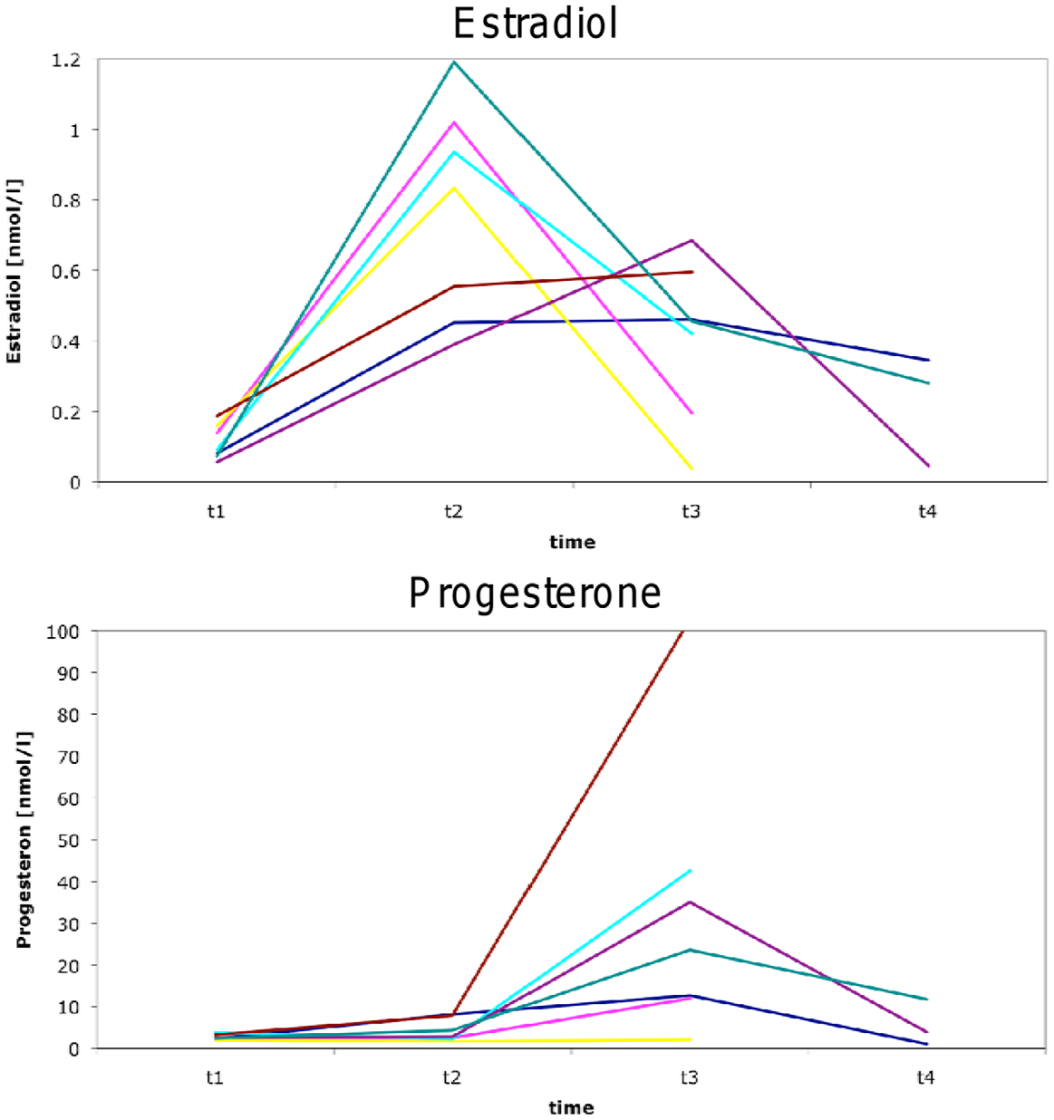
Quantitative hormone changes during the menstrual cycle for estradiol and progesterone of all 8 female subjects. For clarification, time points are connected by straight lines. Note: only half of the subjects were measured at t4; part of the lines overlie. All but one subjects showed hormone levels within physiological levels and indicating ovulatory cycles: This is an increase of estradiol levels at t2 and/or elevated progesterone levels after t2. One subject showed no increase in progesterone at t3, indicating missing ovulation, and was excluded from further studies, along with the matched male.

**Figure 2:**
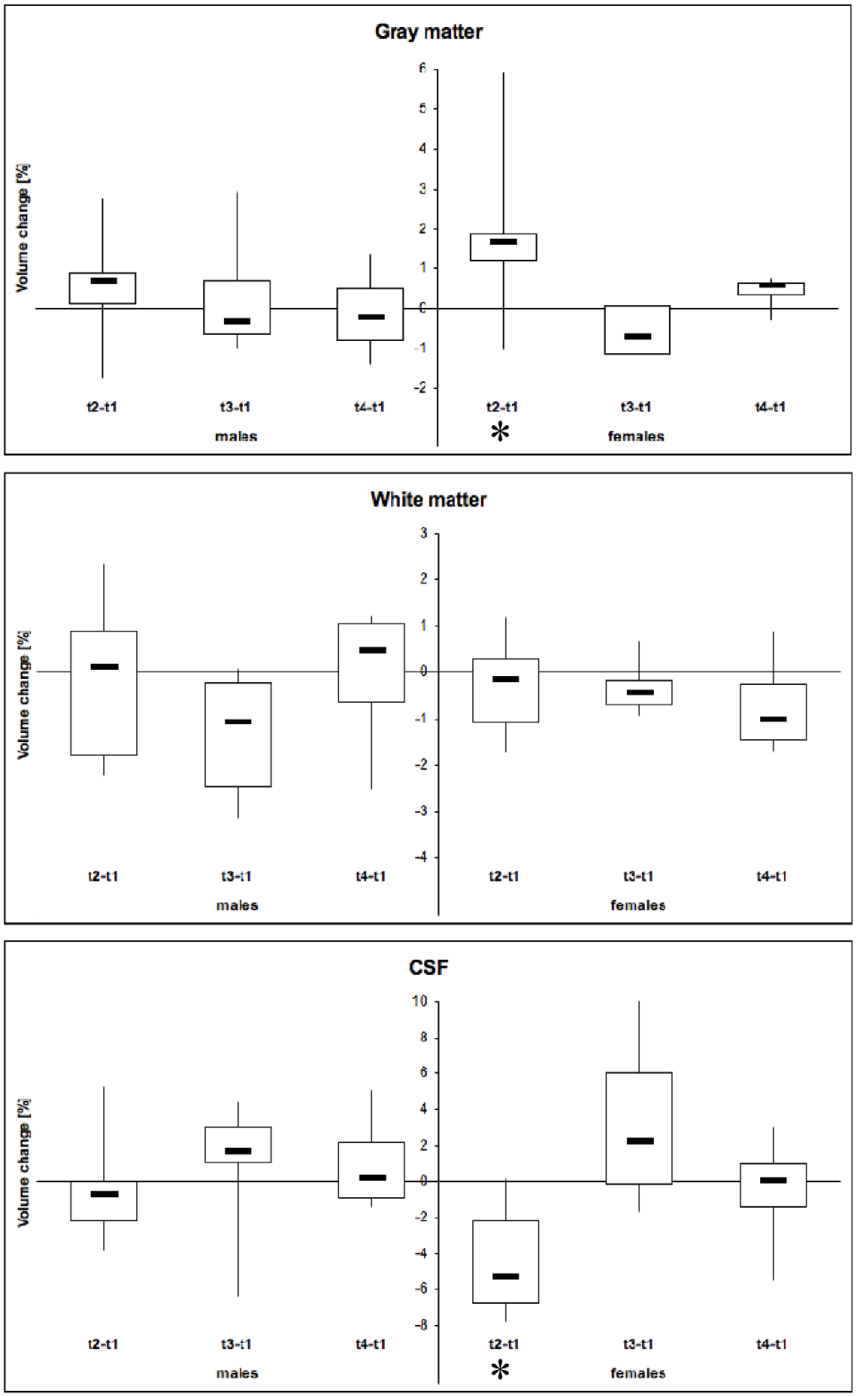
Relative volume change of grey and white matter and CSF between 4 time points during the menstrual cycle in women and in men, respectively. In females (right columns), there is a significant increase in grey matter at the time of ovulation and a corresponding loss of CSF compared to men (p<0.05, asterix). t1: Menses, t2: ovulation; t3 midluteal phase; t4: next menses. The data are displayed as boxplot, where 50 % of data are in the box and whiskers depict range of data. Note: different scale of y-axes.

All but one female showed hormone level modulation during the menstrual cycle which was typical for an ovulational cycle (fig. 1). The time point of ovulation was estimated by ultrasound (t2). One subject showed no increase in progesterone at t3, indicating missing ovulation, and was excluded, along with the matched male. So, data from seven females (age range: 21–31 y) and seven males (age range: 23–37 y) were included in the analysis of volumetric data. In females, GM showed a significant volume increase at ovulation (t2 versus t1) (+1.81 %, p<0.05) and a corresponding volume loss in CSF (−4.4 %; p<0.05) (fig. 2). All WM volume changes and relative volume changes between other time points did not show any significant alterations. In males there was no significant difference in the relative volume changes of WM, GM and CSF during the 4 week period. For a “standard” brain of 1350 ml, the volume changes described in our study allowed for the calculation of a total brain volume increase at ovulation of ∼ 13,5 ml.

**Figure 3:**
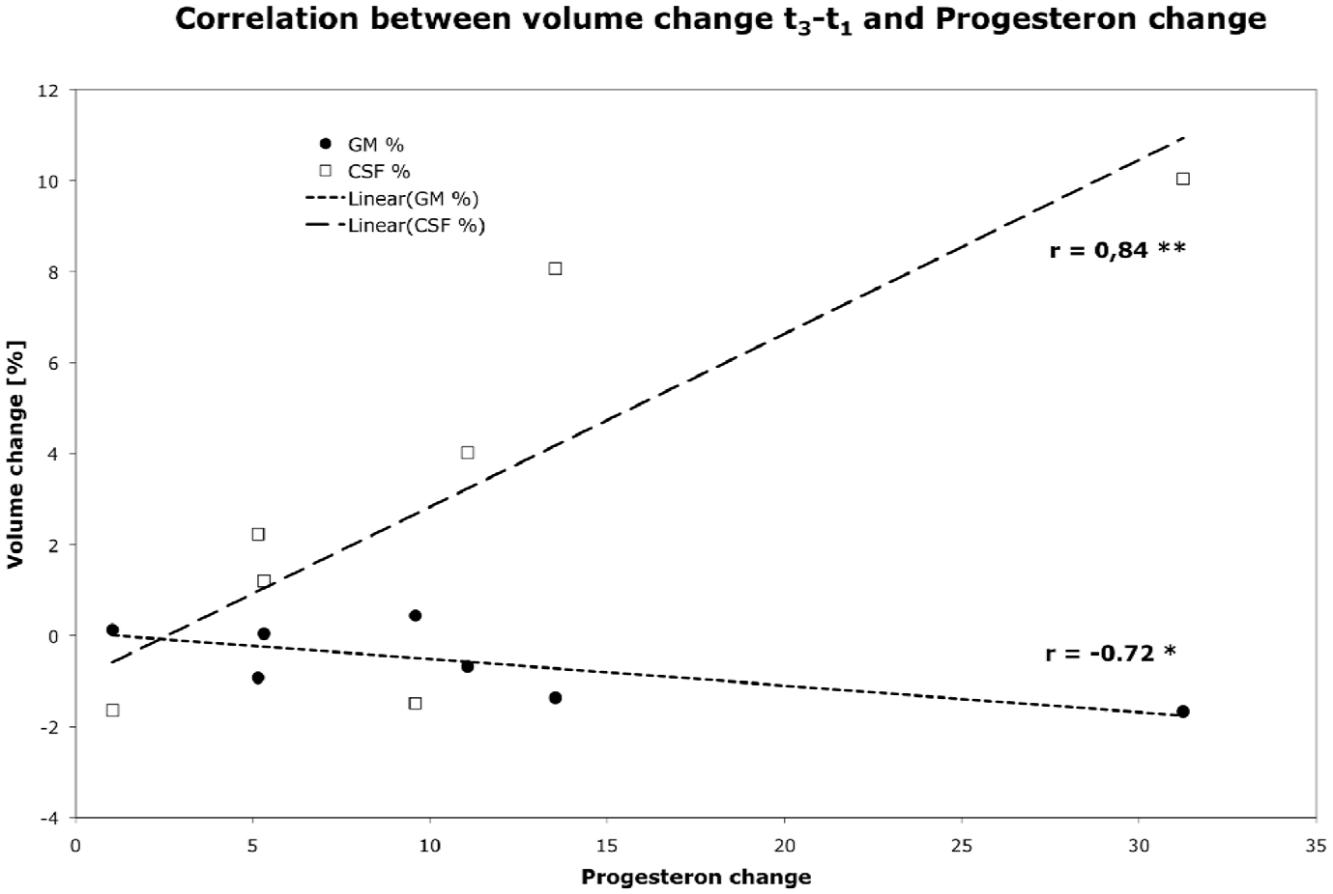
Correlation between the (not significant) relative volume change of grey matter and CSF in females between t3 and t1 and progesterone levels at the appropriate time points.

Although the relative volume changes of GM and CSF between time points t1 and t3 were not significant, these volume changes showed a significant correlation with progesterone changes (fig. 3; GM: r = −0.72; p<0.05; CSF: r = 0.84; p<0.001). There was no statistical correlation between the significant volume changes between t2 and t1, and estradiol changes, which were clearly highest at time of ovulation (t2, see fig. 1).

## Discussion

We demonstrate for the first time that brain morphology varies during the menstrual cycle, with a (grey matter) volume peak at time of ovulation which can be estimated to be ∼13,5 ml for a “standard” brain.

It is tempting to speculate that there is a causal relationship between hormone levels and brain volumes. During the menstrual cycle estradiol levels show a marked peak at ovulation and progesterone levels rise afterwards with a broader peak towards the next menses. However, we could not detect a significant correlation between the grey matter volume peak and the estradiol peak when taken all female subjects into account. On the other hand the brain volume change, and even more so the associated loss in CSF-volume between t1 and t3 which by itself was not significant, correlates well with changes in progesterone levels. In a similar study on pregnant women, Oatridge et al. describe a ∼5 % brain volume loss during pregnancy with a minimal volume at term and a normalization during the few months postpartum [22]. As both estradiol and progesterone levels increase during pregnancy, no conclusions can be drawn as to which hormone contributes to this volume change in the cited study.

We found the lack of correlation between the estradiol rise and the volume changes in our study between t1 and t2 puzzling and, therefore, reanalyzed the data after excluding one statistical outlier (>2SD difference from the mean): Having done this, there was a significant correlation between GM and CSF volumes between time points t1 and t2 and estradiol changes (GM: r = 0.79; p<0.05; CSF: r = −0.76; p<0.05, data not shown) with persistence of a significant grey matter volume peak at time of ovulation. We are well aware that this further reduces our sample size and can only be done with caution. On the other hand this may be a first hint encouraging further, larger studies, to explore this interrelation in more detail.

In the long-term, there are preliminary data suggesting a neuroprotective role for estrogens on age-related temporal atrophy [14]. This effect is most pronounced in postmenopausal women and can be accentuated with hormone replacement therapy [15], [16]. Other steroid hormones like cortisol are also known to decrease brain size long-term [23]. Recently, Cho (2001) demonstrated that even “physiologically” elevated stress induced cortisol levels in cabin crews of long distance flights reveal temporal lobe atrophy associated with decreased memory performance after 5 years of service [24]. Little, however, is known about short-term steroid effects on brain volume. As expected, cortisol levels were stable across the menstrual cycle in our group (data not shown), excluding any confounding effects. Up to now, it is not clear which microscopical mechanisms underlie brain volume changes: contributions can be attributed to changes in vascular supply, intra- or intercellular water shifts, cell hypertrophy, synaptogenesis or even (neuro-)neogenesis [6], [22], [25], [26], [27].

There is compelling experimental evidence that brain structural alteration do occur in adulthood. The formation and turn-over of new neocortical synapsis can be induced in an experience-dependent manner and is probably even increased after injury, hereby facilitating reorganization and recovery of function after injury [27], [28], [29] Hatton and colleagues demonstrated increased dendritic bundling of supraoptic neurons after the induction of maternal behavior [30]. Adult neurogenesis has been identified in all vertebrate species examined thus far [31]. It can be increased or modified by e.g. by exercise, enriched environment, epileptic seizures or during pregnancy [26], [32], [33], [34]. Up to now it is not clear, which functional role newborn neurons play. They may have a beneficial contribution to adaptational processes as well as represent a meaningless side effect [34], [35], [36]. It is, however, only a remote possibility that the amount of new neurons can significantly contribute to macroscopical alterations of brain morphology.

Nevertheless, it is tempting to speculate that mechanisms of synaptogenesis and synaptic rearrangements, which have been described in rodents, also play a role in humans: in rats fast estrous-dependent or estrogen induced changes of spine density have been described in several brain regions including CA1 neurons in the hippocampus and the ventromedial nucleus of the hypothalamus [2], [5], [17], [37], [38], [39], [40]. At least in the hippocampus there is evidence that estrogen not only increases the number of spines per se but also the number of spines with multiple presynaptic boutons ending on different postsynaptic cells [5]. Such network alterations with new connections may have important functional implications, both, in terms of pathophysiology of disease as well as for long-term potentiation (LTP) which is assumed to be a model of learning and memory. They may alter the excitability of the hippocampus, potentially underlying the decrease in seizure threshold seen with estrogen treatment and across the estrous cycle, as well as the estrogen-dependent increment and facilitation of LTP [39], [41], [42]. Although these are rodent studies, they might suggest that synaptogenesis is an underlying mechanism of the grey matter changes observed.

On a functional as well as structural level in humans a vast amount of studies suggest that neuroactive steroids play a role in sexual dimorphism (for rev. [9], [10]. There are clear-cut sexual differences in specific cognitive tasks, in behavior and in the vulnerability to depression and anxiety [11], [43], [44]. Furthermore, memory and learning, pain perception, and susceptibility to seizures are among the CNS functions which are modulated by steroids [45], [46], [47], [48]. Some but not all of these effects show menstrual cycle dependent fluctuations. The diversity of functional CNS-effects, which steroids exert on the brain suggests that they modify widespread brain regions. Besides the classical genomic mechanism by which steroids bind to intracellular receptors [49], it is now clear that they can also act via direct membrane mechanisms, such as ligand-gated ion channels and neurotransmitter transporters, which do not depend on estrogen receptors [50], [51], [52]. This may be an explanation for the widespread actions of estrogens on the brain. In addition, recent work describes estrogen receptors not only on neuronal cells but also on astrocytes and oligodendrocytes [52], [53]. At present, less is known about estrogen and progesterone receptors in the human brain [13], [54], [55]. It might be at hand to conclude that also in the human brain steroid receptors are abundant. However, the wide abundance of steroid effects on the brain gives no further explanation why we found significant volume differences only in grey matter.

Functional imaging studies suggest cycle-dependent changes of activation patterns which are most pronounced for cognitive tasks [1], [56], [57], [58], [59]. They are task and region specific and do not necessarily go along with observational effects [1], [58]. There is uncertainty as to whether hormone levels correlate with cycle-dependent lateralization of specific tasks and spatial extent and/or activation pattern [1], [8], [56], [57], [58]. Changes in the spatial extent of activation can probably be explained by direct vascular effects of estrogen, which is a known vasodilator and could hereby influence brain volume as measured with MRI [60]. The estrogen peak shortly before ovulation could also underlie the brain volume peak, but could not be demonstrated in our subjects before removing one statistical outlier (s. above).

There are several pitfalls of MR-volumetry which have to be considered: physiological parameters like water balance and nutrition may interact with brain volume to a degree which is in the range to be expected with degenerative disease and of the same magnitude we found in our study [25], [61]. However, we minimized these factors by measuring at fixed time points early in the morning with restricted fluid and food intake.

The paucity of conclusive neuroimaging data of hormone effects on brain activity in humans can partly be explained by poor control of hormone levels in most of these studies. On the other hand, might it be a misconception that all sex differences can and have to be explained solely by sex hormones [8]. Our structural observation of cycle-dependent brain volume changes highlights the need to control for phase of the menstrual cycle and hormone levels not only when investigating into function but also in quantitative structure-associated brain imaging, especially in longitudinal studies. It might even be wise to concentrate on either males or females. Although, up to now we can only speculate on what is responsible for the brain volume changes during the menstrual cycle, the changes are too pronounced to ignore them in the future. Furthermore, our results give further evidence of the short-term plasticity of the human brain and it remains to be investigated whether they reflect the structural correlates of cycle-dependent functional states.
